# Health Misinformation in Ethiopia: Myths, Media Dynamics, Public Response, and Policy Implications: A Narrative Review

**DOI:** 10.1002/puh2.70181

**Published:** 2025-12-31

**Authors:** Trhas Tadesse Berhe, Dube Jara, Dereje Kifle

**Affiliations:** ^1^ Frontieri Consult, PLC Addis Ababa Ethiopia

**Keywords:** community mobilization, Ethiopia, health misinformation, myths, public health communication, social media

## Abstract

**Background:**

Health misinformation in Ethiopia undermines public trust and weakens the effectiveness of health interventions. Cultural beliefs, religious influences, and the expansion of digital media contribute to myths that fuel vaccine hesitancy, stigma, and delayed health‐seeking behavior.

**Objective:**

To synthesize evidence on the scope, drivers, and impacts of health misinformation in Ethiopia and to highlight actionable strategies for improving public health communication.

**Methods:**

A narrative literature review was conducted using PubMed, Scopus, and African Journals Online, supplemented with grey literature from the Ministry of Health, World Health Organization (WHO), United Nations Children's Fund (UNICEF), and Regional fact checking organizations. Sources published between 2010 and 2025 that addressing misinformation, communication channels, or public responses in Ethiopia were included. Findings were summarized using descriptive narrative synthesis.

**Result:**

Misconceptions related to traditional remedies, vaccine safety, COVID‐19 cures, and modern contraceptives are widespread. Narratives spread rapidly across social media, particularly Facebook and Telegram, whereas oral traditions reinforce misinformation in rural communities. These Documented impacts include reduced uptake of immunization and maternal services, delayed treatment for diseases such as TB and HIV, and persistent stigma. Interventions involving community health workers, religious leaders, and youth‐led campaigns have proven effective in countering misinformation.

**Conclusion:**

Health misinformation remains a significant barrier to Ethiopia's health targets. Strengthening media literacy, engaging trusted community actors, and building partnerships between government, civil society, and digital platforms are crucial to mitigate health misinformation and improve public health outcomes.

## 1 Introduction

Health misinformation has become a global public health concern, particularly in the digital era where information spreads rapidly across borders and communities [1]. It refers to false or misleading information about health, regardless of intent, which can undermine public trust, reduce adherence to medical recommendations, and compromise disease prevention and control efforts [2]. Globally, health misinformation has been linked to vaccine hesitancy, delayed treatment seeking, and the persistence of harmful health practices [3].

In Ethiopia the challenge is particularly pronounced due to the country's unique sociocultural and linguistic diversity. With over 80 languages spoken, strong religious traditions, and deeply rooted cultural beliefs, health messages often compete with long standing community perceptions [4]. Misconceptions about diseases such as HIV/AIDS, tuberculosis, and maternal health conditions are prevalent. For example, a nationwide study among 11,425 reproductive age women found that 27.5% held at least one misconception about HIV transmission, such as believing it could spread through mosquito bites or sharing food [5]. In some emerging regions, over 70% of sexual active women were found to hold at least one false belief about HIV transmission [6]. Traditional beliefs about illness, such as the evil eye or spiritual punishment, often coexist with modern biomedical explanations, which can sometimes create confusion and reduce trust in the formal healthcare system [7, 8].

The expansion of internet access and social media platforms has introduced new dynamics in misinformation dissemination. Platforms, such as Facebook, Telegram, and TikTok, serve as powerful communication tools and a as major vectors for misinformation [9, 10]. During the COVID‐19 pandemic, for instance, community‐based studies in southern Ethiopia reported that 41%–57% of the residents held misconceptions about transmission and prevention, whereas unverified remedies such as garlic, ginger, or coffee were widely promoted [11, 12]. These misconceptions have contributed to vaccine hesitancy, reliance on traditional remedies, delayed care seeking, and broader public health risks [13, 14].

Health misinformation is generated through multiple pathways, including misinterpretation of scientific findings, anecdotal experience, rumors, and deliberate disinformation campaigns. Psychological factors, such as confirmation bias, fear, and social reinforcement, amplify the spread, whereas social and cultural norms influence which messages gain traction in communities [15].

To address misinformation, Ethiopia has implemented community‐driven strategies involving health extension workers, religious leaders, and youth organizations disseminate accurate health messages [16]. National initiatives, often in collaboration with World Health Organization (WHO) and United Nations Children's Fund (UNICEF), have focus on enhancing communication campaigns and monitoring misinformation trends, strengthening public awareness, and trust in health interventions [17].

Given the *magnitude of misinformation*, its *severe impact on health behaviors*, and the *broader public health implications*, there is a pressing need to understand the myths circulating in communities, the dynamics of media through which misinformation spreads, and public responses to these false narratives (The terms italicized in this paragraph aim to synthesize empirical evidence on health misinformation in Ethiopia, exploring circulating myths, media dynamics, public responses, and policy implications, and identifying strategies to enhance community engagement, strengthen health communication, and inform policy interventions.)

## 2 Methods

This review employed a narrative literature review approach to synthesize evidence on health misinformation in Ethiopia, focusing on the scope, drivers, and public health implications. Although narrative reviews typical do not follow the full systematic review protocol, we incorporated structured search and screening methods to ensure comprehensiveness and transparency. The review was designed to incorporate both peer‐reviewed academic publications and grey literature to ensure comprehensive coverage of the Ethiopian context, including formal research, policy reports, and media analysis. The approach allowed for the integrated understanding of the mechanisms through which health misinformation spreads and its consequences for public health.

### 2.1 Literature Search Strategy

A systematic search was conducted across major electronic databases, including PubMed, Scopus, Web of science, and African Journal Online (AJOL), complemented by grey literature from the Ethiopian Ministry of Health, the WHO, UNICEF, and Africa Check. The review included evidence from local media outlets, policy briefs, and fact‐checking organization to capture various perspectives, offering a comprehensive understanding of health misinformation in the Ethiopian context. The search strategy employed combination of keywords and Boolean operators such as “health misinformation” OR “false health information” OR “health rumors” AND “Ethiopia” OR “Ethiopia public health” OR “myth” OR “traditional beliefs” OR “social media” OR “digital platform” OR “mass media” AND “vaccine hesitancy” OR “COVID‐19 misinformation” OR “HIV stigma.” This comprehensive strategy ensured the inclusion of studies reflecting both digital and community‐level dissemination of misinformation.

### 2.2 Eligibility Criteria

#### 2.2.1 Inclusion Criteria

This review included studies, program reports, and grey literature that were conducted in Ethiopia or reported Ethiopia‐specific findings and focused on health misinformation, myths, rumors, or misinformation affecting the population. Eligible studies addressed topics related to infectious diseases, vaccination, maternal and child health, or health‐seeking behavior; were published between 2010 and 2025 in English or Amharic; and were available in full text.

#### 2.2.2 Exclusion Criteria

Studies were excluded if they focused solely on clinical medicine without relevance to health communication or misinformation, were conducted outside of Ethiopia without Ethiopia‐specific findings, or were unavailable in full text.

### 2.3 Data Extraction and Synthesis

Following the identification of eligible studies, all materials were screened by title and abstract, followed by the full text review to confirm inclusion. Key information extracted included types and sources of misinformation (e.g., traditional myths and social media rumors); channels of dissemination (e.g., word of mouth, digital platforms, religious, or community networks); affected health areas (COVID‐19, HIV/AIDS, TB, reproductive health); reported consequences (vaccine hesitancy, delayed care, stigma); and strategies or interventions used to counter misinformation.

Data extraction was conducted independently by two reviewers, with discrepancies resolved through discussion to ensure accuracy and reliability. Extracted data were then systematically coded into thematic domains and synthesized using narrative thematic analysis. Quantitative findings (e.g., prevalence or frequency of misinformation) were summarized across studies, whereas qualitative insights were integrated to contextualize behaviors, beliefs, and intervention outcomes. Thematic categories included (i) prevailing myths and misconceptions, (ii) the role of media in spreading misinformation, (iii) public health consequences including vaccine hesitancy and maternal health outcomes, and (iv) community and institutional responses. This structured approach facilitated the creation of summary tables (Tables 2–4) and supported a comprehensive, evidence‐based narrative highlighting patterns, gaps, and interventions addressing health misinformation in Ethiopia.

### 2.4 Quality Appraisal

To ensure the credibility of included evidence, all studies were assessed using an adapted version of the Joanna Briggs Institute (JBI) Critical Appraisal Checklist for qualitative, quantitative, and descriptive studies. The appraisal focused on methodological rigor, clarity of reporting, transparency of data collection and analysis, and relevance to the Ethiopian context. Each study was independently evaluated and assigned a quality rating of high, moderate, or low. Only studies meeting acceptable methodological standard were included in the synthesis.

Among the 27 studies included, 20 were rated as high quality and 7 as moderate quality, with no studies receiving a low‐quality rating (Table 1).

**TABLE 1:** Quality Assessment of Studies Included in the Review on Health Misinformation in Ethiopia.

Reference	Study type/Design	Population/Setting	Sample size /Scope	Focus/Topic	Quality rating
Borges do Nascimento et al. [2]	Systematic review of reviews	Global (including Ethiopia)	31 studies	Infodemics and health misinformation	High
Adebesin et al. [1]	Bibliometric analysis	Global	N/A	Social media misinformation during	High
Kunguma [11]	Quantitative survey	Ethiopia	450 participants	COVID‐19 home remedies and myths	Moderate
Assefa et al. [17]	Mixed methods	Ethiopia (community health extension)	National coverage	Community health extension success/challenges	High
Mohammed et al. [18]	Cross sectional	Sekota Zuria Woreda	380 mothers	Colostrum avoidance	High
Aynalem et al. [19]	Qualitative	East Gojjam Zone, Ethiopia	30 participants	Cultural beliefs and practices in pregnancy	High
Burayu and Degefa [20]	Cross‐sectional	Southern Ethiopia	500 mothers	Harmful traditional practices	High
Kalayou and Awol [21]	Community based cross‐sectional survey	North Ethiopia	420 adults	COVID‐19 vaccine	High
Sedlander et al. [22]	Survey/Quantitative	Rural Ethiopia	350 women	Contraceptive myth and infertility fear	High
Estifanos et al. [23]	Quantitative	Ethiopia (two regions)	40 healthcare workers	Data falsification and misinformation	Moderate
Morgan and Wendland [24]	Review	Africa	N/A	Abortion related misinformation	High
Sadore et al. [25]	Online cross sectional	Ethiopia	300 residents	Social media influence on COVID‐19 measures	Moderate
Tadesse et al. [26]	Cross sectional	Addis Ababa, Ethiopia	600 adults	COVID‐19 vaccine hesitancy	High
Sahile et al. [27]	Systematic review/Meta analysis	Ethiopia	N/A	Vaccine acceptance and hesitancy	High
Jarso et al. [28]	Cross sectional	Eastern Ethiopia	450 HCWs	Vaccine side effects and perceptions	High
Kiross et al. [29]	Qualitative	Ethiopia	40 caregivers	Health‐seeking behavior for infants	High
Tesfa et al. [30]	Cross sectional	Gedeo zone Ethiopia	500 women	Unmet maternal health info needs	High
Jember et al. [31]	Community based cross sectional	Gonder, Ethiopia	380 residents	COVID‐19 vaccine	High
Beshah et al. [32]	Scoping review	Ethiopia	N/A	Vaccine hesitancy prevalence	High
Assefa et al. [33]	Mixed methodes	Ethiopia	400 caregivers	Gender dynamics in vaccine acceptance	High
Ebabu and Muhammed [34]	Cross sectional	Afar pastoralist community	250 mothers	Maternal traditional practices	High
Kea et al. [35]	Qualitative	Sidama zone Ethiopia	35 participants	Childbirth misconceptions	High
Workineh et al. [36]	Qualitative	Bahir Dar, Ethiopia	30 participants	Maternal near miss and misinformation	High
Kahissay et al. [37]	Qualitative	North East Ethiopia	25 participants	Sociocultural beliefs of ill health	High
Eyeberu et al. [38]	Cross sectional	Harari, Ethiopia	420 adults	Risk perception and care seeking	Moderate
Akafu et al. [39]	Cross‐sectional	Manna district, Ethiopia	380 CBHI members	Trust in healthcare system	High
Azoulay [40]	Qualitative/Commentary	Ethiopia	N/A	Fact checking in authoritarian context	Moderate
Sadore et al. [41]	Cluster RCT	Ethiopia	20 clusters	Religious leader training and ANc uptake	High
Datiko et al. [42]	Mixed methods	Southern Ethiopia	Multiple communities	Health extension workers and maternal service	High
Negussie et al. [43]	Mixed methods	Ethiopia (national campaign)	N/A	Media campaigns on safe breastfeeding	High

Abbreviation: CBHI, community‐based health insurance.

### 2.5 Study Selection Process

To ensure transparency in documenting the literature selection pathway, elements of systematic search reporting were incorporated. Although this review follows a narrative synthesis approach, the study identification, screening, and inclusion process is illustrated using a PRISMA flow diagram.

A total of 495 records were initially identified from electronic databases (PubMed, Scopus, Web of science, and AJOL) and grey literature sources (Ethiopian Ministry of health, WHO, UNICEF, Africa Check, and local media). After removing duplicates (*n* = 79), 416 records were screened by title and abstract.

**FIGURE 1:**
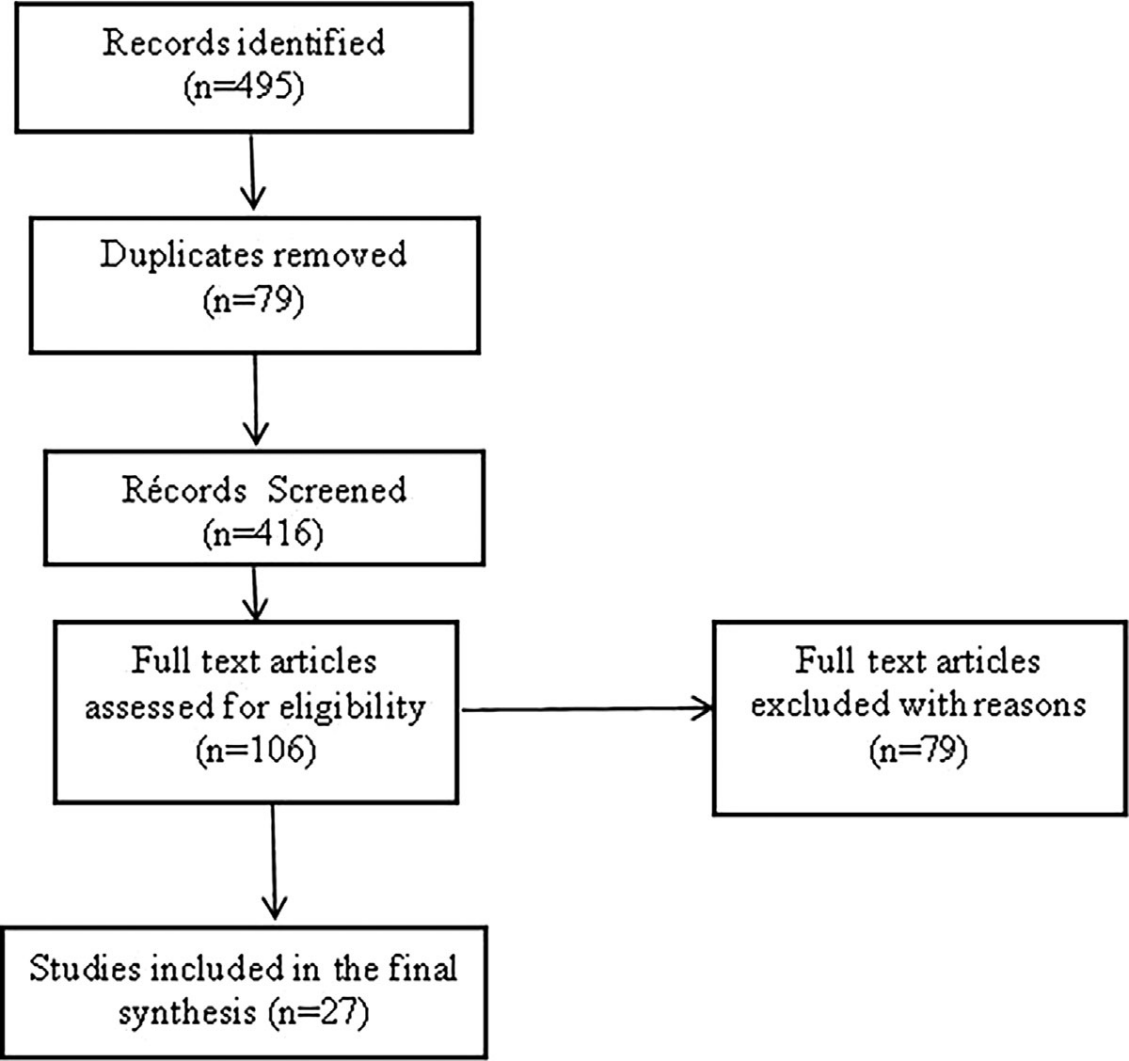
PRISMA Flow diagram for the study selection.

Following screening, 106 full text articles were assesses for eligibility, of which 79 were excluded for not meeting inclusion criteria (conducted outside Ethiopia, unrelated to health misinformation, or not accessible in full text). Finally, 27 studies and program reports were included in the qualitative synthesis (Figure 1).

## 3 Results

A total of 27 studies and program reports met the eligibility criteria and were included in the qualitative synthesis. The majority were cross‐sectional surveys, mixed methods studies, or qualitative inquires conducted in different Ethiopian regions. Evidence was synthesized in to key thematic domains that illustrate the nature of misinformation, channels of information, and associated health outcomes in the Ethiopia context.

### 3.1 Prevailing Health Myths and Misconceptions

Health misinformation in Ethiopia is shaped by a complex interplay of cultural beliefs, traditional practices, and limited access to reliable information, which collectively influence maternal and child health behaviors and decision‐making [13]. For instance, a community‐based study in Sekota Zuria Woreda found that 19% of mothers avoided colostrum feeding due to negative attitudes, insufficient knowledge, and low participation in community health programs, demonstrated how cultural practices and misinformation can directly impact neonatal health outcomes [44]. Similarly, a study in East Gojjam Zone identified practices such as drinking holy water, taking herbal medicine, and adhering to food taboos during pregnancy and child, which despite their cultural significance, may contribute to maternal and neonatal complications in the absence of evidence‐based guidance [19]. Furthermore, research in Southwest Ethiopia revealed that 76.3% of mothers engaged in harmful traditional practices during the puerperal period, with factors like low educational status, rural residency, absence of antenatal care, and home deliveries being significantly associated with these practices, highlighted the role of limited access to healthcare services and information in perpetuating such behavior [20]. Prevailing myths include the perception that COVID‐19 [45] or modern contraceptive method cause infertility [22], and that HIV can be cured through traditional herbs or spiritual practice [5, 6].

These beliefs are often reinforced by anecdotal narratives, religious interpretations, and community gossip [21]. Such misinformation not only shapes community health‐seeking behavior but also contributes to delays in accessing essential services [23, 24, 25] (Table 2).

**TABLE 2:** Summary of Prevalent Health Myths and Misconceptions in Ethiopia.

Health topic	Myth	Source	Evidence summery
COVID 19 [4, 10, 11]	Garlic, ginger, or coffee can cure COVID 19	Social media, community rumor	Community‐based surveys showed significant reliance on anecdotal remedies, contributing to delayed care seeking
Vaccines [22, 30, 31]	TT vaccine or COVID 19 causes infertility	Social media, informal networks	Survey data indicate up to 48% of respondants hold misconceptions about vaccine safety
HIV/AIDS [5, 6, 13, 24]	Can be cured through traditional herbs or spiritual rituals	Community networks, traditional healers	Qualitative interview revealed persistence of beliefs in unproven cures, affecting ART adherence
Maternal health [18, 19, 20, 39]	Facility delivery is unsafe. Certain foods during pregnancy are harmful	Oral traditional, religious interpretations	Cross‐sectional studies report 19%–76% of mothers practicing harmful traditional behavior, impacting neonatal outcomes
Contraceptives [5, 23]	Modern contraceptives cause long‐term infertility	Community gossip, social media	Misconceptions contribute to low contraceptive uptake in reproductive age women
TB [4, 42]	Spiritual punishment or traditional remedies are effective treatment	Community network	Misconceptions delay timely diagnosis and treatment initiation

*Note:* Data in this table were extracted from the included studies and synthesized thematically.

### 3.2 Roles of Media in Spreading Misinformation

Digital platforms, including Facebook, Telegram, TikTok, and WhatsApp, serve as a significant conduits for health misinformation in Ethiopia, enabling unverified claims to circulate more rapidly than official health communications [1, 4]. Evidence shows the significant influence of these media channels on public perceptions. For instance, a community‐based study in Northeast Ethiopia reported that 39.4% of respondents agreed that COVID‐19 vaccine side effects are dangerous, whereas 35.7% believed vaccines contain harmful or questionable substances [30]. Similarly, a survey in Addis‐Ababa found that 57.4% of participants were hesitant or unwilling to be vaccinated, largely due to safety concerns and rumors circulating online and through informal media networks [15]. Collectively, these findings highlight how social and traditional media can both support rapid public health communication and simultaneously amplify fear, uncertainty, and conspiracy narratives, thereby shaping health behaviors and undermining trust in evidence‐based care [2] (Table 3).

**TABLE 3:** Media Channels and Misinformation Spread in Ethiopia.

Media type	% of population exposed	Examples of misinformation	Evidence summery
Facebook [22, 30]	40%–50%	COVID‐19 vaccine side effects exaggerated	Cross‐sectional surveys show correlation between social media exposure and vaccine hesitancy
Telegram [4, 6]	30%	Claims that herbal remedies cure HIV	Community interviews reveal rapid spread of unverified treatment information
TikTok [4, 10]	15%–25%	Misleading COVID‐19 treatment videos, such as miracle cures, unapproved drugs, fake demonstrations, and others	Qualitative studies show short video content drives misconceptions and fear about modern healthcare
WhatsApp [22, 31]	35%–45%	Rumors on fertility effects of vaccines	Evidence suggests peer to peer messaging amplifies myths, particularly regarding reproductive health
Community networks (local social groups influencing maternal and child health beliefs and decisions) [18, 19, 20, 39]	70%–80%	Common misconceptions (inaccurate or unscientific beliefs) about pregnancy and childbirth, such as dietary restrictions, avoiding exercise or chores, beliefs about prolonged labor or rituals, postpartum practices like delaying breastfeeding, fears about contraception, and avoidance of antenatal care or hospital deliveries	Local social groups reinforce traditional beliefs contributing to delayed maternal and neonatal health‐seeking behavior

*Note:* Data in this table were extracted from the included studies and synthesized thematically.

### 3.3 Public Health Consequence of Health Misinformation

Health misinformation suboptimal on in Ethiopia is associated with delayed care seeking, nonadherence to treatment, and maternal and child health outcome. More than 70% of women were reported to have unmet maternal health information needs, with limited access to reliable mass media sources [34]. The circulation of false or misleading information through social media and community networks exacerbates vaccine hesitancy and delayed utilization of essential maternal and child health services [30]. Vaccine hesitancy, in particular, has been significantly influenced by misinformation, resulting in suboptimal immunization coverage, especially among healthcare workers and pregnant women [37]. Despite initiatives such as the expanded program on immunization (EPI) and health extension program (HEP), gaps persist in coverage among pastoralist, conflict‐affected, and remote populations, emphasizing the need to address misinformation as a major barrier to equitable health outcome [17, 33].

#### 3.3.1 Misinformation and Vaccine Hesitancy and Immunization Coverage

Vaccine hesitancy in Ethiopia has been significantly exacerbated by the widespread circulation of misinformation, leading to suboptimal immunization coverage. For instance, a community‐based study in Gonder found that nearly half (48%) of residents held misconceptions about the COVID‐19 vaccine, which directly undermined vaccine uptake and pandemic control efforts [30]. Similarly, national scoping review revealed that vaccine hesitancy exceeded 50% in more than 40% of included studies, with particularly high prevalence among healthcare workers (69.7%) and pregnant women (68.8%) [17]. Together, these findings demonstrated that misinformation amplified by inadequate health promotion and limited access to reliable information has become a major barrier to immunization programs and maternal health protection in Ethiopia [44]. Despite the expansion of the EPI and the HEP, substantial coverage gaps persist among pastoralists, conflict‐affected, and hard‐to‐reach populations in Ethiopia. Childhood immunization coverage is projected to increase from 14.4% in 2000 to 53.6% by 2025; however, this remains far below the 90% target outline in the immunization agenda 2030 (IA2030), with misinformation emerging as a major barrier to equitable vaccine uptake [45].

#### 3.3.2 Maternal Health Misinformation and Adverse Outcome

Misinformation remains critical determinants of adverse maternal outcomes in Ethiopia. In the pastoralist communities of Afar, nearly one third of women (32.8%) reported adhering to nutritional taboos during pregnancy, whereas 17%–18% practiced abdominal massage behavior often driven by misconceptions about promotes maternal and fetal health, there by increased risk of complications [19]. Similarly, qualitative research in Sidama revealed that misconceptions surrounding childbirth safety, the perceived superiority traditional birth attendants, and limited maternal health knowledge contributed to delay in seeking skilled care [20]. Recently, a maternal near‐miss study in Bahir Dar highlighted misinformation, low decision‐making capacity, and continued trust in traditional practices as community‐level contributors to severe maternal morbidity [39]. Collectively, this evidence highlights the magnitude of the problem and the urgent need for culturally responsive, evidence‐based interventions to counter misinformation and reduce preventable maternal morbidity and mortality in Ethiopia. Recently, a maternal near‐miss study in Bahir Dar highlighted misinformation, low decision‐making, and continued trust in traditional practices as community‐level contributors to maternal morbidity [39]. Collectively, this evidence highlighted the magnitude of the problem and the urgent need for cultural responsive, evidence‐based interventions to counter misinformation and reduce preventable maternal morbidity and mortality in Ethiopia.

#### 3.3.3 Misinformation and Health‐Seeking Behavior

Misinformation significantly shapes health‐seeking behavior in Ethiopia, particularly in rural and marginalized communities. Evidence from East Gojjam indicated that many caregivers fail to recognize the severity of childhood illness or delay seeking care due to misconceptions about symptoms and reliance on traditional healers [17]. Nationally, nearly one in two caregivers relies on home‐based or traditional remedies before seeking professional care, often resulting in delayed treatment and preventable complications [44]. During the COVID‐19 pandemic, misinformation amplified this challenge, with community surveys in Harari showing that only 35.6% of adults reported willingness to seek healthcare when needed, whereas 50.6% held low risk perceptions of the diseases [30]. These patterns show that misinformation not only erodes trust in modern health services but also contributes directly to avoidable morbidity and mortality by delaying timely access to life saving interventions.

#### 3.3.4 Misinformation and the Erosion of Trust in the Health System

The spread of misinformation erodes trust in the health system and drives communities to rely on informal or traditional sources of advice rather than professional guidance. A recent WHO systematic review of 31 studies concluded that infodemics and misinformation significantly undermine health behaviors by increasing vaccine hesitancy, delaying care seeking, and amplifying use of unproven treatments [2, 39]. In Ethiopia, community‐based surveys showed that only 35.6% of adults in Harari were willing to seek healthcare during COVID 19, whereas 50.6% demonstrated low risk perceptions of the diseases, largely shaped by misinformation [39]. Furthermore, studies on community‐based health insurance (CBHI) members in Ethiopia revealed that perceived poor quality of care and lack of trust in health facilities reduced enrollment and service utilization (*β* = −0.47, 95% CI: −0.64, −0.29) [39]. This erosion of trust, amplified by social media‐driven misinformation, creates a self‐perpetuating cycle in which formal health services are bypassed, weakening diseases prevention and health promotion efforts.

In general, misinformation has profound and multidimensional consequences on maternal and child health in Ethiopia. Addressing these issues requires a multifaceted approach.

### 3.4 Community and Institutional Responses

Efforts to combat health misinformation in Ethiopia are multilayered, involving governmental, nongovernmental, and community‐based initiatives. Although international fact checking organizations, such as Africa Checks, have provided guidance and resources across Africa, local fact checking capacity in Ethiopia remains limited, with emerging initiatives like HaqCheck and Africa fact checking fellowship beginning to address misinformation [9]. Community engagement programs that strategically leverage religious leaders, health extension workers, and local influencers have demonstrated substantial and measurable improvements in maternal and child health outcomes in Ethiopia. A recent cluster randomized trial found that training religious leaders to provide maternal health education increases antenatal care attendance by 21.4%, institutional deliveries by 20%, and postnatal care utilization by 22.3% compared with control communities [25]. Similarly, targeted interventions involving health extension workers through the health development army and pregnant women's forums enhanced participation in maternal health programs, leading to increased identification and referral of pregnant women and improved utilization of antenatal and skilled delivery services [46]. These findings emphasized the critical role of cultural integrated community actors in promoting maternal health service uptake and highlighted the effectiveness of context‐specific engagement strategies in low resource settings.

Public health campaigns in Ethiopia increasingly employ multimedia strategies integrating radio, television, and social media with culturally tailored messaging to maximize reach and impact. For example, national wide media campaign promoting safe breastfeeding practices during the COVID‐19 pandemic reached approximately 50% of target mothers and general public and significantly increased awareness and perceptions regarding the importance of continuing safe breastfeeding [43]. Similarly, studies in rural eastern Ethiopia indicated that access to health messages via mass media remains critical with exposure to radio, television, social media, and printed media significantly increased the likelihood of receiving and acting on health information [45].

Nevertheless, significant barriers persist, such as low digital literacy, especially in rural households; limited internet and media infrastructure in remote areas; and deeply rooted traditional beliefs continue to shape health behavior and challenge the uptake of verified information [47, 48, 49]. These realities highlighted the urgent need for sustained and context‐sensitive interventions that strengthen local fact checking capacity, foster community trust improved digital literacy, and adapt health communication to cultural content (Table 4).

**TABLE 4:** Summary of Community and Institutional Interventions in Ethiopia.

Intervention	Target group	Outcome	Evidence summery
Religious leaders training on maternal health [18, 41]	Pregnant women and community	20%–22% in antenatal care institutional deliveries, postnatal care, increase maternal health service utilization and referrals	Cluster RCT showed culturally integrated engagement improved uptake of maternal health services
Health extension workers and health development army [17]	Pregnant women and caregivers	Increase maternal health service utilization and referrals	Program evaluations indicate measurable improvements in maternal health service utilization
National media campaigns (radio TV, social media) [16, 43]	General public, mothers	Reached 50% of target population, increased awareness of safe breast feeding	Survey report improved knowledge and perception change during COVID 19
Community engagement programs [45, 46]	Rural community	Enhanced trust in health services and uptake of verified health information	Mixed methods studies highlight positive effects of community led initiatives on maternal and child health behavior

*Note:* Data in this table were extracted from the included studies and synthesized thematically.

## 4 Discussion

The findings of this review highlight the pervasive and multifaceted nature of health misinformation in Ethiopia. Cultural norms, religious practices, and limited access to reliable information collectively reinforce harmful beliefs that compromise maternal and child health. The evidence indicates that misinformation not only delays care seeking but also contributes to vaccine hesitancy, low immunization coverage, and reliance on unproven remedies, consistent with global observations of infodemics during pandemics [1, 2, 3]. Local studies further show that misinformation regarding HIV/AIDS, COVID‐19 vaccines, and maternal health is widespread, reflecting the role of entrenched traditional beliefs and low health literacy [4, 5, 22].

Digital and social media are powerful tool for both dissemination of accurate information and propagation of myths. The widespread circulation of misinformation highlighted the urgent need for tailored health communication strategies that consider local cultural contexts, literary level, and media access studies have shown that low digital literacy and limited access to reliable media exacerbate the spread of false health narratives [8, 30, 35, 47]. Intervention leveraging community actors such as leaders and health extension workers have proven effective, emphasizing that culturally integrated approaches are essential in low resource setting [17, 37, 42].

The review also emphasizes the interplay between misinformation and trust in health systems. Communities exposed to persistent false narratives are less likely to utilize formal health service, which in turn perpetuates health disparities. Addressing these challenges requires a multipronged strategy combining digital literacy, evidence‐based health promotion, continues rumor monitoring, and supportive policy frameworks [1, 41, 50]. Strengthening trust trough transparent communication and engagement with local stakeholders is critical to enhance service uptake and health equality.

Future research should focus on evaluating the effectiveness of intervention aimed at curbing misinformation and improving health outcomes across diverse Ethiopian context [1, 50]. Particularly attention should be given to integrating digital health tool, culturally tailored messaging, and community‐based approaches, as evidence suggests these strategies significantly enhance health literacy and behavioral change [1, 7, 47]. Rigorous studies, including randomized trials and implementation research, are essential to identify best practices for scalable, sustainable interventions.

In conclusion, health misinformation in Ethiopia poses a substantial threat to maternal and child health. Comprehensive, context sensitive strategies that integrate community engagement, digital literacy, and cultural tailored message are critical to mitigate the negative consequence of misinformation and strengthen public health outcomes. Collaboration among public health authorities, media platforms, and local leaders is necessary to ensure timely dissemination of evidence‐based information and to build resilient health systems capable of countering misinformation [17, 37, 47].

## 5 Limitations

This review has several inherent limitations. It relies on narrative synthesis because the included studies varied widely in design, population, and outcomes, making quantitative pooling inappropriate. As a result, the findings are descriptive and may not reflect the full statistical scope of health misinformation effect. Only published evidence in English and Amharic was included, potentially overlooking evidence in other local languages. The review primarily draws from published literature, which may under‐represent informal or community‐based sources of misinformation. Rapidly evolving digital media and variability in study contexts may also affect the generalization of the findings.

## 6 Conclusion and Future Directions

Health misinformation remains a significant public health concern in Ethiopia driven by cultural beliefs, social network, and rapid expansion of digital media.

Addressing this challenge requires culturally sensitive, multilevel strategies that strengthen community engagement, improve health communication, and enhance digital health literacy. Collaboration between public health authorities, media institutions, fact checking organizations, and trusted local influencers is essential to promote timely dissemination of accurate health information and build public trust. Future efforts should include continuous monitoring of emerging myth, evaluate of misinformation prevention interventions, effectiveness, and prioritize outreach in rural and marginalized communities to enhance public health resilience and improve maternal and child health outcomes.

## Author Contributions

All authors agreed on the content of the article and contributed significantly to its development. Trhas Tadesse Berhe led the conceptualization, design, data analysis, and drafting of the manuscript. Dube Jara and Dereje Kifle contributed to the study design, literature review, data interpretation, and critical revision of the manuscript. Trhas Tadesse Berhe is the guarantor of the work. All authors reviewed and approved the final version of the article.

## Ethics Statement

The authors have nothing to report.

## Consent

The authors have nothing to report.

## Conflicts of Interest

The authors declare no conflicts of interest.

## Patient and Public Involvement

Patients and/or the public were not involved in the design, or conduct, or reporting, or dissemination plans of this research.

## Data Availability

No datasets were generated or analyzed during the current study.
